# Auditory-induced body distortions in children and adults

**DOI:** 10.1038/s41598-020-59979-0

**Published:** 2020-02-20

**Authors:** Elena Nava, Ana Tajadura-Jiménez

**Affiliations:** 1 Department of Psychology, University of Milano-Bicocca, Piazza dell’Ateneo Nuovo 1, 20126 Milan, Italy; 2 DEI Interactive Systems Group, Department of Computer Science and Engineering, Universidad Carlos III de Madrid, Madrid, Spain

**Keywords:** Sensory processing, Human behaviour

## Abstract

Recent studies have shown that body-representations can be altered by dynamic changes in sound. In the so-called “auditory Pinocchio illusion” participants feel their finger to be longer when the action of pulling their finger is paired with a rising pitch. Here, we investigated whether preschool children - an age group in which multisensory body-representations are still fine-tuning - are also sensitive to this illusion. In two studies, sixty adult and sixty child participants heard sounds rising or falling in pitch while the experimenter concurrently pulled or pressed their index finger on a vertical (Experiment 1) or horizontal axis (Experiment 2). Results showed that the illusion was subjected to axis and age: both adults and children reported their finger to be longer in Experiment 1, but not in Experiment 2. However, while in adults the feeling of finger elongation corresponded to a recalibration of the fingertip’s felt position upwards, this was not the case in children, who presented a dissociation between the feeling of finger elongation and the perceived fingertip position. Our results reveal that the ‘auditory Pinocchio illusion’ is constrained to the vertical dimension and suggest that multisensory interactions differently contribute to subjective feelings and sense of position depending on developmental stage.

## Introduction

A number of studies, mostly performed in adults, have shown that the mental body representation is a dynamic one, and is continuously updated by multisensory cues (see^1–3^ for recent reviews). Neuroimaging and neurophysiological studies have shown that the brain receives information about the body from the eyes, the ears, the skin, and these signals are integrated in frontal, parietal and temporal convergence zones in the cortex^4,5^. Evidence that such multisensory interactions shape body representations comes from experiments in which body illusions were used; here, multisensory cues are commonly put into conflict to show that the brain responds to such conflict by creating distorted perceptions of the body. One of the most cited body illusions is the ‘rubber hand-illusion’, in which the synchronous stroking of the participant’s hand (hidden from the participant’s view) concurrently with a visible life-sized rubber hand induces the participants to perceive the fake hand as theirs^6^. The illusion emerges because of the fusion of visual, tactile and proprioceptive cues - that is, the synchronous stroking changes the proprioceptive and tactile representations of the hand, favouring the match between visual and proprioceptive information, thus leading to the feeling that the (rubber) hand I am seeing is actually my own. Evidence that this illusion is not only driven by visual input but is rather a genuine multisensory phenomenon comes from studies that have observed that the illusion also occurs in the absence of visual feedback, such as in the ‘somatic’ version of the illusion^7,8^. Interestingly, the ‘rubber-hand-illusion’ only occurs when the rubber hand is placed in an anatomically plausible position^9,10^, which suggests that the illusion is modulated by top-down signals originating from the visual representation of one’s own body (see also^1,11^).

While it is difficult to isolate and quantify the contribution of the single sensory modalities to body illusions, as they are inherently multisensory in nature, there are body illusions in which one sense clearly dominates over the others in driving the changes in bodily perception. For instance, seeing a body improves tactile spatial resolution at that specific body part^12,13^, reduces the perceived size of touch, as assessed using the mirror box illusion^14^, and ameliorates tactile deficits^15^. Similarly, proprioception influences body representation in body illusions involving muscle vibrations. This is the case of the famous ‘Pinocchio illusion’: by applying a vibrating force to the biceps or triceps tendons of participants who concurrently touch their own nose, the common perception is to feel one own’s nose as elongating^16,17^.

Finally, also audition can alter body representation. Although the influences of this modality on body perception have been less investigated than those from other senses, there are growing studies documenting that sound alters the perception of bodily experiences. For instance, it was shown that the modulation of the amplitude of sounds while subjects concurrently rub their two hands together changes their perception of smoothness or dryness of the skin^18^. Furthermore, Senna and colleagues^19^ showed that by gently hitting the participants’ hand with a small hammer, while progressively changing the sound of the hammer as if it was hitting marble, lead participants to perceive their hand harder and stiffer as if it turned into marble. Other studies have shown that manipulating the sound feedback of one’s own footsteps to make it consistent with the footstep sounds of lighter or heavier bodies, leads to changes in represented body size/weight^20,21^; or that artificially lengthening the time it takes to hear the impact of an object being dropped from one’s hand on the ground, leads people to feel taller^22^.

Audition can also alter tactile distances related to perceived arm length, as shown in a study by Tajadura-Jimenez and colleagues^23^, in which participants were asked to tap on a surface with their hand while extending their arm sideways, and concurrently heard a tapping sound which originated at different distances. When the distance between the hand location and the sound doubled, participants reported increased tactile distance on the arm, as assessed on a two-alternative forced-choice tactile distance perception task. That is, the discrepancy between the perceived location of the sounds and the hand location altered the perceived length of the arm of the participants. This occurred without any conscious awareness, as participants did not report to feel their arm as longer. Follow-up studies with this paradigm provided evidence that consciously perceived changes in arm-representation can be evoked by the manipulated tapping sounds that relate to the changes in perceived tactile distance^24^. These studies also showed that such sound alterations can lead to performing reaching actions as if one’s arm was indeed longer^25^.

In the past years, there has been growing interest in the development of body representation, its earlier and later development^8,26,27^. In particular, a series of studies have addressed whether children are susceptible to bodily illusions too, particularly using the rubber hand illusion.

Interestingly, sensitivity to some characteristics of bodily illusions appear to change throughout development. For example, in the classical rubber hand illusion, studies have shown that children of different ages report the fake hand to be theirs; this occurs in the visual version of the illusion^26,27^, in the somatic version^8^ and in the full-body version^28^. However, there appears to be developmental changes that occur both at the level of subjective feeling of owning the rubber hand and the proprioceptive drift, a typical measure of shift in hand-centred reference frame. For example, as reported by Cowie and colleagues^26,27^, from the age of 6 years, children reliably report the subjective feeling of owning the rubber hand. However, this is not the case in younger children (4 to 5 years of age). Furthermore, children’s proprioceptive drifts were larger than adults’, suggesting that between 4 and 9 years of age, children’s hand position may be more affected by viewing a fake hand and this pattern may decline between childhood and adulthood.

The fact that children do not present adult-like proprioceptive drift is suggestive of the possibility that children are yet not able to fully integrate multisensory information. Indeed, in order to self-locate one’s own hand towards the rubber hand, one needs to integrate the sensory modalities, thus fusing the tactile stimulus with the seen (rubber) hand and the position of one’s own hand. However, several developmental studies have shown that multisensory integration is a late bloomer^29^, extending up to 8–10 years of age, which in turn appears to explain why children are also insensitive to bodily illusions. This is particularly the case of multisensory optimal integration, by which the resulting percept is a weighted average of the individual sensory estimates with weights proportional to their precision. Children are not able to optimally integrate sensory information to reduce uncertainty for different sensory combinations, be it visuo-haptic^30,31^ or audio-haptic combinations^32^.

Evidence that children’s multisensory integration abilities is absent in younger children and does not emerge before later childhood has also been corroborated by studies investigating multisensory facilitation in motor responses, in which participants are required to provide fast responses to unisensory or multisensory stimuli. While adults commonly benefit from multisensory signals that cannot be explained by coactivation models (i.e., Race Model, see^33^), children display multisensory facilitation that is consistent with coactivation, i.e., no multisensory integration^34^.

Overall, these studies suggest that multisensory integration undergoes a protracted development and may well underlie the development of body representation too, as revealed in adult studies showing the association between body representation and multisensory processing^2,35^.

To date, however, studies have addressed children’s bodily illusion using particularly the rubber hand illusion^8,26,27^ and the full-body illusion^28^, which all share the interaction of different sensory modalities, such as vision, proprioception and touch, but have not investigated the influence of audition and its interaction with other sensory modalities in the development of body representation. Indeed, no study has tested other illusions in children, and no illusion in which the auditory domain was involved.

Thus, in this study, we took advantage of a newly reported audio-haptic illusion, the ‘auditory Pinocchio’ illusion^36^, to observe whether children are also sensitive to this illusion. Tajadura-Jimenez and colleagues^36^ reported that when adult participants pull on their index finger while presented with brief sounds of rising pitch, they have the feeling that their finger is getting longer. In this illusion, the auditory stimulus is represented by a sound that changes in pitch, which is primarily determined by frequency, but several studies have shown that the cognitive system maps pitch onto a mental representation of space. Indeed, it has been documented that high pitches tend to be mapped to high positions in space, while low pitches tend to be mapped to low positions in space across different domains [ref. ^37^; for a recent review, see^38^). While such mapping between dynamic pitch changes and vertical motion has been mostly demonstrated across audition and vision, it has been demonstrated that it also applies across audition and touch^39^. According to a study by Parise and colleagues^40^, sounds coming from high elevations have more energy at high frequencies, which parallels the filtering properties of the outer ear. Thus, it is likely that pitch-elevation mapping may reflect the tuning of the auditory system to the statistics of the natural sounds. Furthermore, this natural adaptation may have been reinforced by universal use of spatial terms for describing pitch; for instance, pitch is labelled as being ‘high’ or ‘low’ in different languages, and the idea of space as a metaphor has also been used to refer to different aspects of music and musical experience (e.g., the tonal space).

Interestingly, the spatial nature of pitch appears to be sufficient to induce the auditory Pinocchio illusion, suggesting that metaphoric sounds that are not indicative of veridical movement (i.e., a rising pitch) can induce changes in body representation when arbitrarily paired with a bodily action.

Recent developmental studies have provided evidence that not only preschool children spontaneously match rising and falling sounds to visual objects moving along the same dimension (i.e., vertical, see^41^), but even 3-month-old infants prefer ‘metaphoric’ correspondences between audio-visual compounds^42^. Thus, this predicts than even children may be sensitive to the auditory Pinocchio illusion, but to what extent audition may influence their body representation is the main objective of this study.

Here, we aimed at investigating for the first time the role of sound in body perception in the developmental population using a body illusion that has never been tested before in children, which also consists of novel multisensory combinations (applied to the body), i.e., audio-tactile interactions.

Because the auditory Pinocchio illusion makes use of auditory stimuli that create the illusion of moving in space, this is also the first study that aims at observing whether crossmodal ‘metaphoric’ correspondences influence body perception in children.

We tested children aged 4 to 6 because of two main reasons: first, this particular age group is the youngest in which adult paradigms can be reliably tested. Likely because of this, many studies have targeted this age group (see the different studies by Cowie and colleagues, and Nava and colleagues); thus, testing the same age group allows to make some comparisons and predictions based on previous studies. Second, as mentioned earlier, there is evidence that preschool children are unable to integrate sensory information, and by testing 4 to 6 year-olds, we observed the children who are almost certainly not good at integrating multisensory information, thus making the prediction that if multisensory interactions are crucial for the development of body representation, we would observe constraints on the sensitivity to the illusion in children but not adults.

We conducted two experiments. In Experiment 1, we tested children aged 4 to 6 years of age and adults on the ‘auditory Pinocchio illusion’, in which participants’ index finger was either pulled or pressed by an experimenter along a vertical dimension while they concurrently heard a rising, falling or constant sound. If participants prove sensitive to the illusion, they would report their finger to feel longer selectively in the condition in which the experimenter pulls the participant’s finger while he/she hears a rising pitch. Along with a self-report, we also measured the recalibration of finger position, hypothesising that if the illusion also contributes to more implicit characteristics of body perception (i.e., the body schema), participants would recalibrate their finger position sense upwards with respect to the real position of their finger. In particular, given the impaired multisensory integration processes in children documented in other studies^30–32^ we expected two possible outcomes: on the one hand, if children tend not to integrate audio-haptic information, this will reflect on the mechanisms underlying the illusion, thus preventing them from perceiving the illusion; on the other hand though, studies have shown that children up to 6 years of age present an auditory dominance^43^, and this may reflect on this illusion too, thus promoting perception of the illusion.

In Experiment 2, we wanted to observe how low-level components typically characterising crossmodal correspondences (in this case the co-occurrence of a sound changing in pitch and tactile stimulation to the finger) interact which other higher-level components (in this case body posture) in the binding of multisensory bodily information driving the illusion of finger elongation. To this aim, we tested a new sample of children and adults in which we presented the same stimuli as in the first experiment, but had participants’ arm placed along a horizontal axis, with hands facing rightward. We reasoned that if sounds have a spatial nature, by which high pitches are commonly associated with higher spatial position, and lower sounds to lower spatial positions, placing the arm along a horizontal axis would influence how individuals feel the illusion, likely diminishing the strength of it. While changes in pitch are indicative of displacement of objects in the horizontal plane (i.e., ‘the Doppler effect’) evidence shows that horizontal representations of pitch seem to be less automatically mapped and appear to be much more dependent on task and perceiver’s experience (e.g., musicians vs. non-musicians; see^38,43,44^).

Thus, given that none of our participants were musicians, we expected them to be less influenced by the illusion when the stimuli were displayed along a direction that does not match their ‘natural’ axis.

## Method

### Experiment 1

#### Participants

Thirty children between 4 and 6 years of age (14 females, mean age = 4.80, SD = 0.66) and 30 adults between 19 and 27 years of age (23 females, mean age = 24.03, SD = 1.50) took part in this study. Six additional children were tested but not included in the final sample because they did not want to finish the experiment or did not understand the task. All participants had no neurological or cognitive impairments. Six adults and 7 children reported to be left-handed. The children were recruited and tested in kindergartens around the city of Milan (Italy) after both parents provided informed consent. The children received a colourful certificate following the study with their name written on it as a thank you for their participation.

The adults were mostly students and recruited both at the University of Milano-Bicocca and through word of mouth. The students received course credits for their participation. All adults signed an informed consent before being tested. All participants were naïve to the purpose of the study. The study was approved by the Ethics Committee of the University of Milano-Bicocca and conducted following the ethical principles of the Declaration of Helsinki for human testing.

#### Apparatus and stimuli

**Figure 1:**
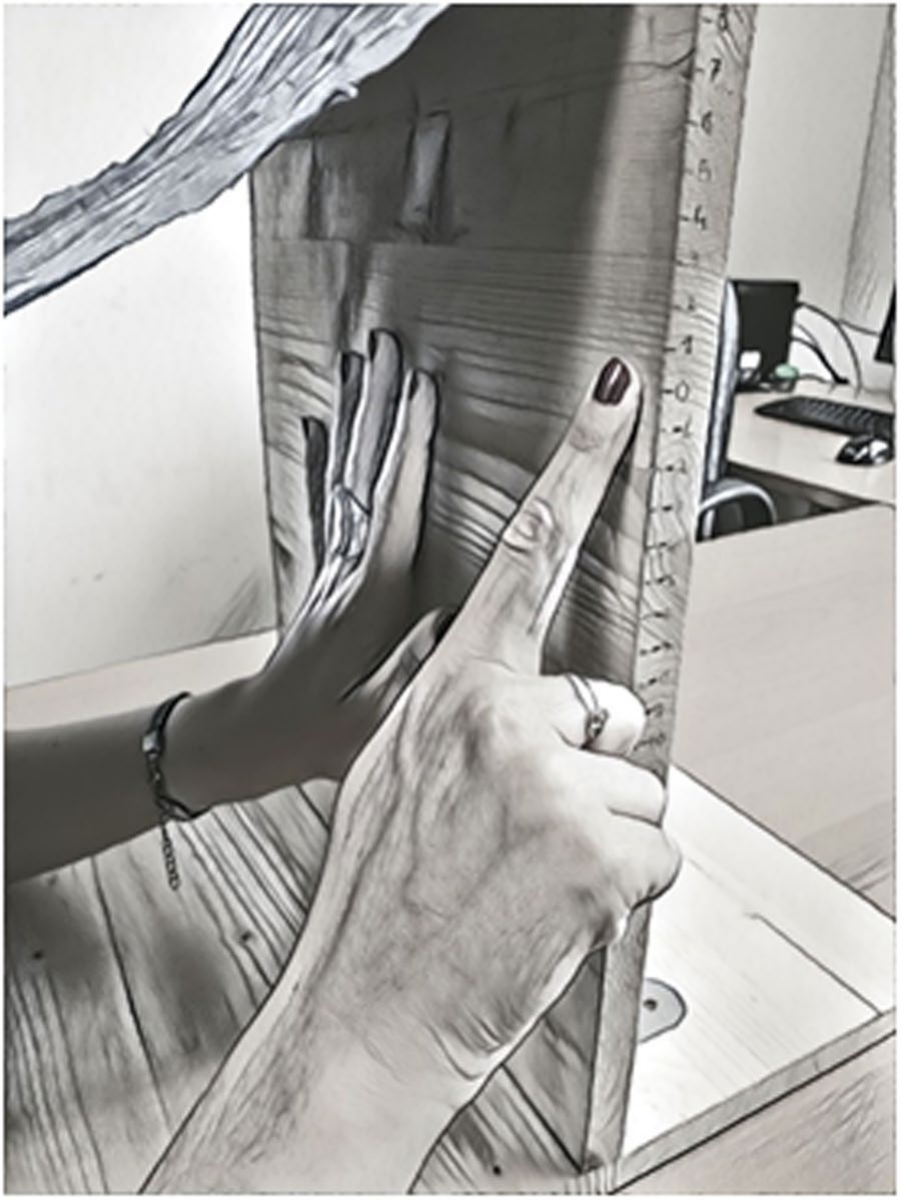
Graphical representation of the set-up and pointing task in Experiment 1.

The experimental set-up is illustrated in Fig. 1 and consisted of a wooden board (40 × 30 cm) attached to a second horizontal wooden surface. Participants sat in front of this board and held the non-dominant hand pressed against the board with the fingertips pointing upwards. A black cloak was attached to the participant’s neck on one extremity and attached at the top of the vertical board on the other extremity to prevent the participant to see her own hand.

The auditory stimuli were similar to the ones described in^36^ and consisted of pure tones (3000 ms duration and 44.1-kHz sample rate) of increasing (‘ascending’ tone: 700 to 1200 Hz), decreasing (‘descending’ tone: 700 to 200 Hz) or constant (‘constant’ tone: 700 Hz) frequency. To prevent clipping, a 10 ms onset/offset ramp was added in the auditory stimuli, which were presented at a 60 dB sound level. The stimuli were generated through Matlab (version 2019a, The MathWorks Inc, Natick, Mass), and presented through headphones. Tactile stimuli consisted in having the experimenter either pulling or pressing the participant’s middle finger.

#### Experimental procedure

Verbal instructions about the tasks were provided to all participants before starting the experiment. In particular, to make sure that participants (particularly children) were able to discriminate the stimuli, we presented them unimodally. That is, we presented them with the three different combinations of tones and tactile stimuli, after each of which we asked them to report what they heard and felt. All participants were able to say whether the sound was ascending, descending or constant, and whether they felt their finger being pulled or pressed by the experimenter. Note that, contrary to^36^, the participants’ finger was pulled or pressed by an experimenter rather than asking the participant to self-touch their own finger. This was motivated mostly by providing a type of stimulation similar across participants and particularly to prevent children from being distracted by this manual task rather than by the illusion. Although we did not control for the pressure applied to the finger of the participant by the experimenter, it is worth noting that only two trained experimenters performed the tactile stimulation, and this allowed us to constrain the differences in pressure. Furthermore, the experimenters were blind to the experimental condition to avoid being influenced by it, and a t-test run between each condition and experimenters revealed no significant difference between experimenters.

Participants were then told that in each trial they would be presented with one of the three tones, concurrently with the tactile stimulus, and that their task was to point to the position in which they felt was the fingertip of their middle finger. Because the whole hand was covered by the black cloak, participants were required to point rightwards (or leftward if left-handed) with respect to their left hand, directly on the wooden board, with eyes open (see Fig. 1). Numbers ranging between −15 cm and +15 cm were drawn directly on the wooden surface (only visible to the experimenter), so that at each pointing, a second experimenter could record the pointing, which was repeated for three times. Note that the “0” corresponded to the tip of the middle finger of the participant, and the pointing directed above the tip corresponded to a positive number, while the pointing directed below the tip corresponded to a negative number.

The experiment started with participants performing 5 pointing movements to indicate their middle fingertip position without any auditory stimulus. This was made to ensure that any potential differences between groups were not due to differences in pointing abilities. After this, participants were presented with all the sensory combinations of auditory and tactile stimuli, i.e., ascending sound with pulling or pressing the finger; descending sound with pulling or pressing the finger; constant sound with pulling or pressing the finger. The combinations were presented randomly, and each combination was presented for 5 times in a row. After each presentation, the participants had to point to the position of their own middle fingertip. At the end of the 5 trials for each combination, the experimenter asked the participants: “Did you feel your finger get longer? and “Did you feel your finger get shorter?”, and participants answered these questions using a 5-point-Likert scale, with −2 corresponding to “not at all”, −1 to “not really”, 0 to “I do not know”, +1 to “a little bit”, +2 “very much”. Note that participants were allowed to take short breaks after each combination if they felt tired.

**Figure 2:**
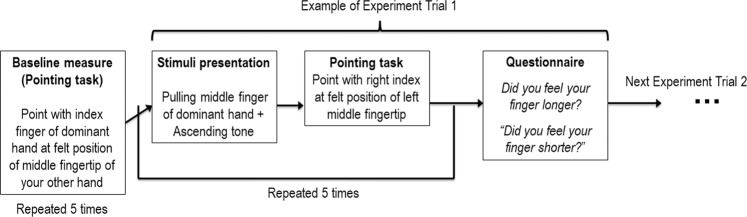
Graphical representation of the experimental procedure of both Experiment 1 and 2.

The experiment took on average 30 minutes to complete, including breaks between conditions (see Fig. 2 for a graphical representation of the procedure).

## Results

### Pointing

The statistical analyses were performed using IBM SPSS (version 25), and the graphs were obtained through GraphPad Prism 8 (GraphPad Software, San Diego, CA).

For each participant we averaged the 5 pointing trials within each combination and entered them as dependent variable in a repeated measures analysis of variance (ANOVA), with Sound (ascending, descending and constant) and Finger (pull and press) as within-subjects variables, and Group (adults and children) as between-subjects factor.

Note that the data were normally distributed in both children and adults, as assessed using Shapiro-Wilk test.

The analysis revealed a main effect of Finger, *F*(1, 58) = 47.83, *p* < 0.001, *ηp*^2^ = 0.45, a Group × Finger interaction, F(1, 58) = 4.78, *p* = 0.03, *ηp*^2^ = 0.08, and a three-way interaction between Sound, Finger and Group, *F*(2, 116) = 12.72, *p* = 0.03, *ηp*^2^ = 0.06. To further explore the three-way interaction, we conducted two further analyses: one to investigate the effects of sound when the finger was pulled (i.e., elongation); the other to investigate the effect of sound when the finger was pressed (i.e., shortening), as also done in the study by^36^.

**Figure 3:**
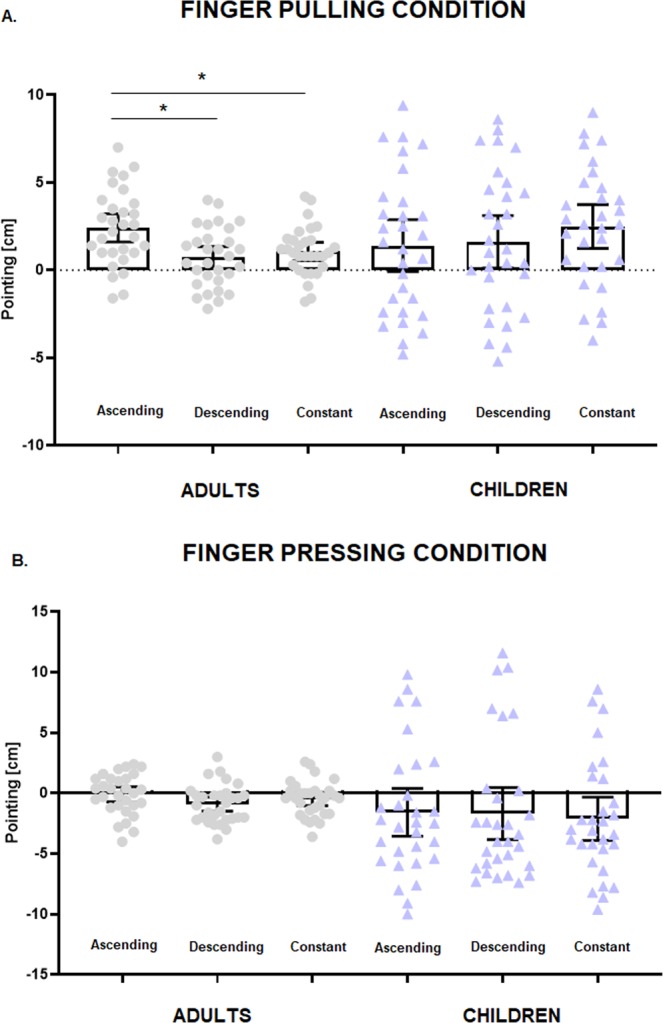
Mean distribution of individual pointing responses of adults and children in Experiment 1, in the condition in which the finger was pulled concurrently with either an ascending, descending or constant sound (**A**), and in the condition in which the finger was pushed concurrently with either an ascending, descending or constant sound (**B**). Error bars represent 95% confidence interval of the mean.

In the analysis made to assess elongation effects on the finger by sound, pointing data was entered as dependent variable in a repeated measures ANOVA, with Sound (ascending, descending and constant) as within-subjects factor and Group as between-subjects factor. We found a Sound × Group interaction, *F*(2, 116) = 6.35, *p* = 0.003, *ηp*^2^ = 0.10, caused by adults presenting larger upward pointing when the sound was ascending than when it was either descending (*p* = 0.01) or constant (*p* = 0.04, Newman-Keuls post-hoc tests), that is, the (congruent) combination of feeling their finger being pulled upwards, while hearing a rising sound, made adults recalibrate their sense of finger position more upwards than its real location. On the contrary, children did not present any difference across conditions (all *p* > 0.14), i.e., the sound did not shift the sense of their finger position in the direction of the sound (see Fig. 3A).

In the analysis made to assess the shortening effects on the finger by sound, pointing was entered in a repeated measures ANOVA with Sound as within-subjects factor and Group as between-subjects factor. Unlike the previous analysis, we did not find any main effect or significant interaction within or between groups (all *p* > 0.40, see Fig. 3B).

### Questionnaire

In order to investigate both the main effects and the interaction between the factors Sound (3 conditions), Question (2 conditions) and Group (2 conditions) we conducted non-parametric analyses of variance (ANOVA) on aligned rank transform data using ARTool (Wobbrock, Findlater, Gergle, & Higgins, 2011). In case of significant effects, these were followed by t-tests between conditions, with the p-value adjusted with the recommended Tukey method for comparing a family of estimates.

**Figure 4:**
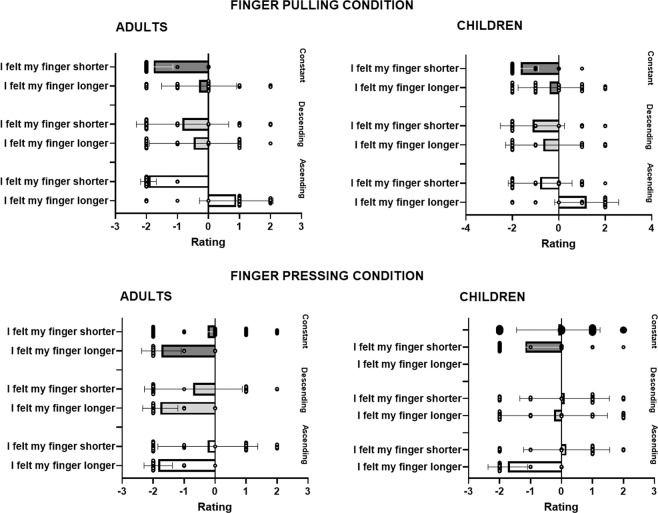
Mean individual responses provided to the questionnaire in Experiment 1 for adults and children in the condition in which the finger was pulled concurrently with either an ascending, descending or constant sound **(A**), and in the condition in which the finger was pushed concurrently with either an ascending, descending or constant sound (**B**). Error bars represent 95% confidence interval of the mean.

To see whether children and adults subjectively felt the illusion, and as done for the pointing, we analysed separately the responses to the conditions when the finger was pulled (i.e., to observe whether the participants felt their finger longer) and to the conditions when the finger was pressed (i.e., to observe whether the participants felt their finger shorter). In the first analysis (i.e., when the finger was pulled), the answers to the questions *“Did you feel your finger get longer”* and *“Did you feel your finger get shorter”* were entered into aligned ranks transformation ANOVAs with Sound (ascending, descending, constant) and Question as within-subjects factor and Group as between-subjects factor. We found a main effect of Sound, *F*(2, 116) = 23.24, *p* < 0.001, *ηp*^2^ = 0.28, due to participants providing more positive ratings when the sound was ascending than when it was descending (*t*(116) = 5.55, *p* < 0.001) or constant (*t*(116) = 6.21, *p* < 0.001); a main effect of Question, *F*(1, 58) = 90.05, *p* < 0.001, *ηp*^2^ = 0.61, due to participants providing more positive ratings after question *“Did you feel your finger get longer”*; and a Sound × Group interaction, *F*(2, 116) = 6.80, *p* = 0.002, *ηp*^2^ = 0.10, due to children providing more positive ratings when the sound was ascending than when it was descending, irrespective of type of question (*t*(116) = 3.83, *p* = 0.028, see Fig. 4A). We also found a Sound × Question interaction, *F*(2, 116) = 24.10, *p* < 0.001, *ηp*^2^ = 0.29, caused by both children and adults providing higher ratings in the illusion question *(“Did you feel your finger get longer?”)* when the sound was ascending than in any other sound condition, and than in response to the question *“Did you feel you finger get shorter”*, for which the response to the ascending sound gave the lowest ratings (all *p* < 0.007), thus revealing that both children and adults reported the illusion of having a longer finger when the sound was ascending.

In the second analysis (i.e., when the finger was pushed) we found a main effect of Sound, *F*(2, 114) = 3.78, *p* = 0.026, *ηp*^2^ = 0.06, due to participants providing higher ratings when the sound was ascending than when it was descending (*t*(114) = 2.56, *p* = 0.031); a main effect of Group, *F*(1, 57) = 12.99, *p* < 0.001, *ηp*^2^ = 0.19, due to children providing overall higher ratings; a main effect of Question, *F*(1, 57) = 32.50, *p* < 0.001, *ηp*^2^ = 0.36, due to participants providing more positive ratings after the second question; and a Sound × Group interaction, *F*(2, 114) = 6.84, *p* = 0.001, *ηp*^2^ = 0.11, caused by children giving higher ratings when the sound was ascending in comparison to all the other conditions, but adults giving higher ratings when the sound was descending (follow-up t-tests were non-significant). There was also a Sound × Question interaction, *F*(2, 114) = 7.67, *p* < 0.001, *ηp*^2^ = 0.12, due to significant differences between the responses provided in the two questions. Follow-up t-tests revealed that for the question *“Did you feel your finger get longer?”*, participants gave higher ratings after the ascending sound than after the descending sound (*t*(227) = 3.33, *p* = 0.013); with other differences between conditions not reaching significance. However, note that the responses to the question *“Did you feel your finger get longer?*” were all below zero (i.e., participants rejected the feeling of having a longer finger), and the responses to the question *“Did you feel your finger get shorter”* were not different from zero (all t-test against zero > 0.12). Thus, in this condition, both children and adults did not report the sense of having a shorter finger (see Fig. 4B).

### Interim discussion

In Experiment 1 we showed that the sensitivity to the finger-length illusion is influenced by age and type of manipulation. Indeed, we were able to replicate the findings of^36^ in young adults, thus showing that even if an adult’s finger is pulled by an external source, the illusion is still perceived, as assessed through implicit (i.e., the pointing task) and explicit measures (i.e., the questionnaire). However, while the illusion of finger elongation seems quite robust, this is not the case with the finger-shortening illusion, as neither adults nor children proved sensitive to this kind of manipulation. Why can the brain ‘accept’ a longer finger but not a shorter one? This asymmetry could reflect the fact that the brain experiences, throughout development, the enlargement of most parts of the body, while it never experiences shrinkage. The fact that even children did not feel their finger to shorten corroborates this interpretation and suggests that even children’s brains are able to keep track of their bodily changes in size.

Interestingly, the auditory Pinocchio illusion appears to undergo developmental changes, as observed in preschool children, who presented similarities and differences to the adult pattern depending on type of stimulation. In other words, while children did not prove sensitive to the finger-shortening illusion, as adults, they presented a dissociation between the explicit and implicit measures of the finger-elongation illusion. Indeed, children reported to feel that their finger was longer when they heard the ascending sound, but they did not recalibrate the felt position of their finger upwards with respect to the real position of their finger. This dissociation is in line with other developmental studies investigating bodily illusions in children^8,45^, and also other adult studies^22,46^, suggesting that, irrespective of type of body illusion, there are often observed differences between self-reports and implicit measures.

In Experiment 1, one of the assumptions of the illusion was that higher pitches are usually associated with higher spatial positions, and lower pitches are associated with lower spatial positions. This metaphoric correspondence, which has been documented both adults^40,43^ and children^41^, appears to facilitate both adult and children to at least subjectively report the finger length-illusion. Interestingly, Experiment 1 also revealed that the ‘metaphoric’ decreasing pitch is not sufficient to make participants perceive their finger to be shorter, and we took this as suggestion that experience with one’s own body growing (and not shrinking) can constrain the perception of the illusion. So, in other words, Experiment 1 appeared to show that congruent, even metaphorical multisensory information promotes bodily illusions, but once you introduce knowledge/experience (i.e., knowing that your body has grown but not shrunk) this prevents the binding of the multisensory information and thus the perception of the illusion.

In Experiment 2, we wanted to further investigate how strong crossmodal correspondences are in influencing the illusion. In other words, by tilting the arm 90 degrees, we created a spatial mismatch between the metaphorical sound (i.e., going up) and the position of the finger (i.e., going rightward).

Would participants still be sensitive to it if their arm was rotated by 90 degrees? In other words, if the same stimuli were presented in the horizontal axis, would participants still report to feel their finger longer? Previous studies have shown that horizontal representations of pitch seem to be less automatically mapped than vertical representations of pitch^38,43,44^, and that bodily illusions related to hand (i.e. the ‘rubber-hand-illusion’) is susceptible to changes in hand orientation^9,10^. We addressed this question in Experiment 2, in which we tested a new sample of both children and adults, with the hypothesis that if audio-haptic correspondences influence the illusion, they both would show less sensitivity to the illusion.

## Experiment 2: Horizontal Condition

### Method

#### Participants

Thirty children between 4 and 6 years of age (17 females, mean age = 4.97, SD = 0.49) and 30 adults between 19 and 26 years of age (23 females, mean age = 23.27, SD = 1.97) took part in this study. Three additional children were tested but not included in the final sample because they did not want to finish the experiment or did not understand the task. Five adults and 6 children reported to be left-handed. Recruitment of participants, as well as inclusion criteria were the same as in Experiment 1.

Note that the parents of all children reported that their children did not receive any formal musical training prior to being tested, e.g., playing an instrument or any musical introductory course. Similarly, also the adults reported not to have experience playing any musical instrument.

#### Apparatus and stimuli

**Figure 5:**
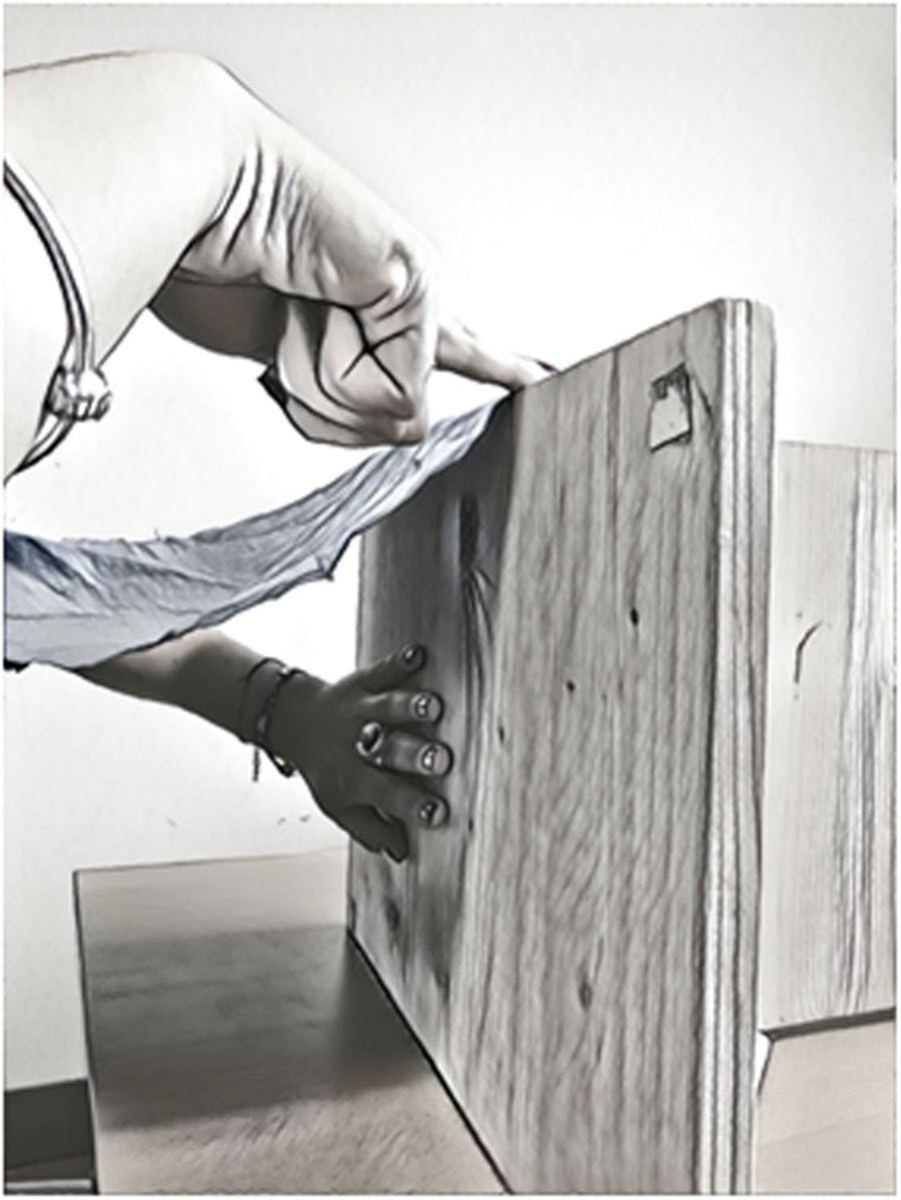
Graphic representation of the set-up and pointing task in Experiment 2.

The experimental set-up was identical to the one used in Experiment 1, with the only difference that we turned the platform on which the hand was placed 90° to its right, so that also the hand was placed with the fingers pointing rightward. Again, as in Experiment 1, a black cloak was attached to the participant’s neck on one extremity and attached at the top of the horizontal board on the other extremity to prevent the participant from seeing their own hand (see Fig. 5). The auditory and tactile stimuli were identical to Experiment 1, with the only difference that the pulling and pressing of the participants’ finger was done rightward and not upward.

#### Experimental procedure

The procedure was similar to the one followed in Experiment 1, with the following differences: first, the pointing was made on the horizontal plane; second, as depicted in Fig. 5, participants were asked to point perpendicular with respect to their other hand. While this manipulation introduces the possibility that participants might have slightly crossed their hands while performing the task, thus making the localisation task more difficult (see^47,48^, but also^49^ for different results), it should be noted that this set-up prevented participants from touching their own hand while performing the pointing, to maintain visual feedback during the task, and finally to maintain a similar distance between hands with respect to Experiment 1.

### Results

#### Pointing

The analyses performed in Experiment 2 mimicked the ones done in Experiment 1. The factors Sound (ascending, descending and constant) and Finger (pull and press) were entered in a repeated measures ANOVA as within-subjects variables, and Group (adults and children) as only between-subjects factor. As in Experiment 1, Shapiro-Wilk normality test showed that the data of both children and adults were normally distributed.

**Figure 6:**
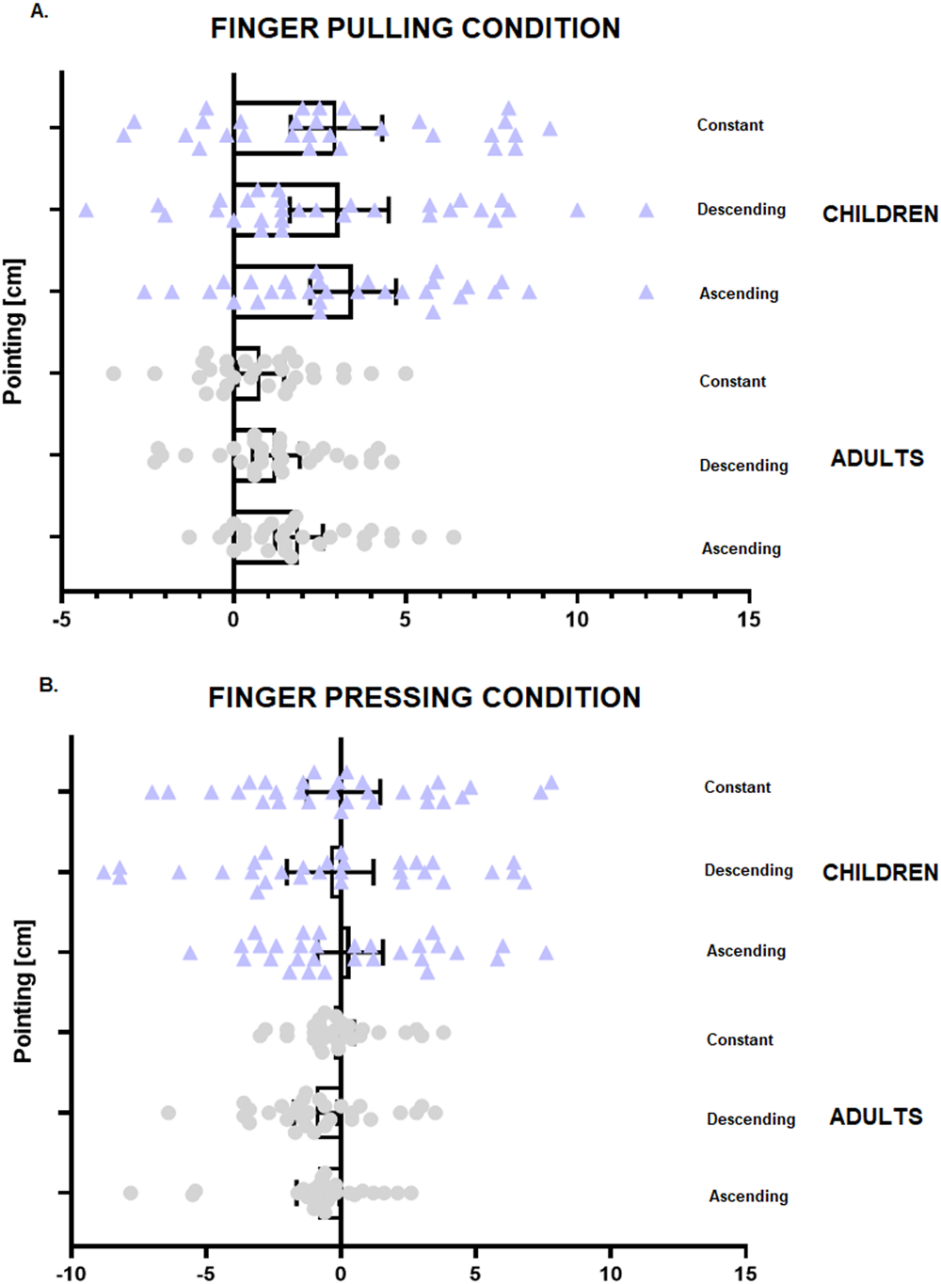
Mean distribution of individual pointing responses of adults and children in Experiment 2, in the condition in which the finger was pulled concurrently with either an ascending, descending or constant sound (**A**), and in the condition in which the finger was pushed concurrently with either an ascending, descending or constant sound (**B**). Error bars represent 95% confidence interval of the mean.

This analysis revealed a main effect of Group, *F*(1, 58) = 4.75, *p* = 0.03, *η*^2^ = 0.08, caused by children displaying overall larger pointing than adults (p = 0.03). There was also a main effect of Finger, *F*(1, 58) = 49.69, *p* < 0.001, *η*^2^ = 0.45, because all participants displayed larger pointing when the finger was pulled, but not when it was pressed, and a Sound × Finger interaction, *F*(2, 116) = 3.88, *p* = 0.02, *η*^2^ = 0.07. However, this interaction was due to all participants making larger rightward pointing irrespective of type of sound presented when their finger was pulled (all *p* < 0.001); that is, sound did not influence the perceived length of the finger, as all participants appeared to be dominated by the tactile modality in this condition (see Fig. 6A,B). Furthermore, note that when the finger was pressed, the participants’ pointing responses did not differ from zero (all *p* > 0.12).

#### Questionnaire

As in Experiment 1, we conducted non-parametric aligned ranks transformation ANOVAs, with significant effects followed by t-tests between conditions, with the p-value adjusted (Tukey method). Further, as in Experiment 1, we conducted two separate analyses in the condition in which the finger was pulled or pressed.

In the condition in which the finger was pulled, we found a main effect of Sound, *F*(2, 116) = 14.95, *p* < 0.001, *η*^2^ = 0.20, due to participants providing higher ratings when the sound was ascending than when it was descending (t(116) = 5.13, p < 0.001) or constant (t(116) = 4.21, p < 0.001); a main effect of Group, *F*(1, 58) = 20.98, *p* < 0.001, *η*^2^ = 0.27, due to children providing higher ratings than adults; a main effect of Question, *F*(1, 58) = 88.19, *p* < 0.001, *η*^2^ = 0.60, due to participants providing higher ratings in response to the question *“Did you feel your finger get longer?”* than “*Did you feel your finger get shorter?*”; and interactions between the following factors: a Sound × Question interaction, *F*(2, 116) = 8.97, *p* < 0.001, *η*^2^ = 0.13, a Group x Question interaction, *F*(1, 58) = 6.76, *p* = 0.012, *η*^2^ = 0.10, and a Sound × Question × Group interaction, *F*(2, 116) = 5.12, *p* = 0.007, *η*^2^ = 0.08. To better explore this three-way interaction, we conducted separate analyses for each group to see whether children and adults perceived the illusion.

**Figure 7:**
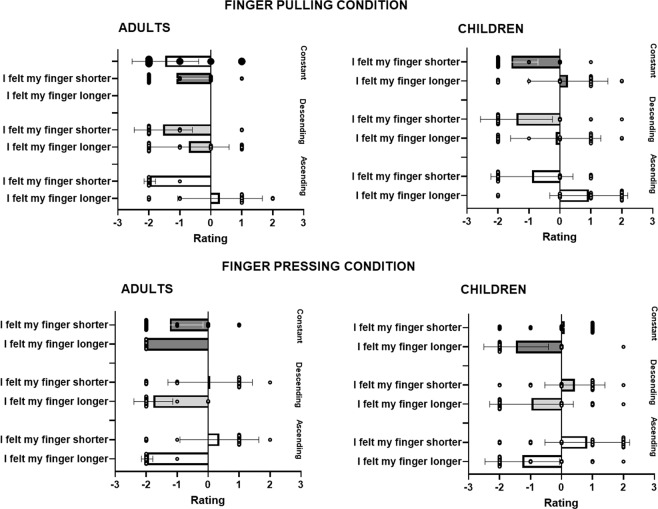
Mean individual responses provided to the questionnaire in Experiment 2 for adults and children in the condition in which the finger was pulled concurrently with either an ascending, descending or constant sound (**A**), and in the condition in which the finger was pushed concurrently with either an ascending, descending or constant sound (**B**). Error bars represent 95% confidence interval of the mean.

In adults, we found a main effect of Sound, *F*(2, 58) = 12.31, *p* < 0.001, *η*^2^ = 0.29, due to participants providing higher ratings when the sound was ascending than when it was descending (t(58) = 3.09, p = 0.008) or constant (t(58) = 4.91, p < 0.001); a main effect of Question, *F*(1, 29) = 59.59, *p* < 0.001, *η*^2^ = 0.68, due to participants providing higher ratings after the first question; and a Sound × Question interaction, *F*(2, 58) = 19.04, *p* < 0.001, *η*^2^ = 0.40, this caused by significantly higher ratings in response to the ascending sound for Question 1 than for Question 2 (t(85.7) = 4.48, p < 0.001), as well as a significant difference in response to the constant sound for the two questions (t(85.7) = 3.56, p = 0.008). However, the ratings to the question “*Did you feel your finger get longer?”* in response to the ascending sound were not different from zero (*p* = 0.23). That is, adults did not report the illusion in this Experiment (see Fig. 7A).

In children, we found a main effect of Sound, *F*(2, 58) = 12.92, *p* < 0.001, *η*^2^* = *0.31, caused by children reporting higher ratings when the sound was ascending in comparison to the descending sound (t(58) = 4.93, *p* < 0.001) and the constant sound (*t*(58) = 3.54, p = 0.002) irrespective of question; and a main effect of Question, *F*(1, 29) = 42.73, *p* < 0.001, *η*^2^ = 0.60, because of children reporting higher responses to the question “*Did you feel your finger get longer*” with respect to the question “*Did you feel your finger get shorter*”, irrespective of sound. However, the lack of a significant interaction between Sound and Finger (*p* > 0.18) suggests that children did not report the sense of having a longer finger in response to the ascending sound (see Fig. 7A).

In the condition in which the finger was pressed, we found a main effect of Sound, *F*(2, 116) = 27.94, *p* < 0.001, *η*^2^ = 0.33, due to participants providing lower ratings when the sound was constant than when it was ascending (t(116) = 6.07, p < 0.001) or descending (t(116) = 6.82, p < 0.001); a main effect of Group, *F*(1, 58) = 19.74, *p* < 0.001, *η*^2^ = 0.25, due to children providing more positive ratings than adults; a main effect of Question, *F*(1, 58) = 127.93, *p* < 0.001, *η*^2^ = 0.69, due to participants providing less negative ratings after the second question; a Sound × Question interaction, *F*(2, 116) = 21.53, *p* < 0.001, *η*^2^ = 0.27. We also found a Sound × Question × Group interaction, *F*(2, 116) = 10.98, *p* < 0.001, *η*^2^ = 0.16. To further explore this interaction we conducted two separate analyses for children and adults.

In adults, we found a main effect of Sound, *F*(2, 58) = 31.51, *p* < 0.001, *η*^2^ = 0.52, due to participants providing lower ratings when the sound was constant than when it was ascending (t(58) = 6.17, p < 0.001) or descending (t(58) = 7.41, p < 0.001); a main effect of Question, *F*(1, 29) = 77.44, *p* < 0.001, *η*^2^ = 0.73, due to participants providing higher ratings after the second question; and a Sound × Question interaction, *F*(2, 58) = 26.55, *p* < 0.001, *η*^2^ = 0.48. This interaction was due to adults providing more negative responses to the question “*Did you feel your finger get longer*” than to the question “*Did you feel your finger get shorter*”. However, also the illusion question “*Did you feel your finger get shorter*” in the presence of a descending sound did not prove different from zero (*p* = 0.12). Thus, adults did not report the finger-shortening illusion (see Fig. 7B).

In children, we found a main effect of Sound, *F*(2, 58) = 4.68, *p* = 0.013, *η*^2^ = 0.14, due to participants providing lower ratings when the sound was constant than when it was descending (t(58) = 3.01, p = 0.01); a main effect of Question, *F*(1, 29) = 55.22, *p* < 0.001, *η*^2^ = 0.66. due to participants providing higher ratings after the second question; and a Sound × Question interaction, *F*(2, 58) = 26.55, *p* < 0.001, *η*^*2*^ = 0.48. This interaction was due to significant differences in the responses to the question “*Did you feel your get finger longer*” after the constant sound than after the descending sound. There were no significant differences between sound conditions for the illusion question “*Did you feel your finger get shorter*” (all ps > 0.8). Thus, children proved to be insensitive to this illusion as adults (see Fig. 7B).

## Discussion

In this study we assessed, for the first time, the role of auditory input on body representation in the developmental population by using the so-called auditory Pinocchio illusion^36^ and addressed whether crossmodal ‘metaphoric’ correspondences influence the sensitivity to the illusion by manipulating the direction in which the finger was either pulled or pushed (i.e., along the vertical and horizontal dimension).

In Experiment 1, we found that children were only partially sensitive to the auditory Pinocchio illusion. That is, they felt their finger longer when pulled by the experimenter while hearing a concurrent ascending sound, but failed to recalibrate their finger beyond their actual finger position. On the contrary, adults reported their finger to be longer and also presented a recalibration towards upper parts of their real finger’s position. This dissociation between subjective reports and position sense appears to be a default at this age, as suggested by other studies that have investigated multisensory bodily illusions in children and have found similar dissociations^8,26,45^. The fact that children are sensitive to the illusions *per se*, but fail in implicit tasks that usually involve a motor output (as in our pointing task; see^8,45^ for similar tasks) suggests that multisensory interactions, necessary to perceive the illusions, differently contribute to what is termed the ‘body image’ and the ‘body schema’. It has been claimed that the body image reflects the thoughts and feeling of one’s own bodily perception, while the body schema reflects a more unconscious sense of body position, mostly used for spatial organisation of action^50^. In our study, the index of the body image was the questionnaire, i.e., the subjective feeling of the illusion, while the body schema was the pointing, which is a motor task. Thus, our data suggest that the body image is already adult-like in early childhood, while the body schema still needs to develop.

It should be noted that dissociations between reported sense of self and body position have been reported for adults too. For instance, Rohde and colleagues^46^ assessed the rubber hand illusion in a group of adults but, instead of measuring self-reports of sense of body ownership and recalibration of position sense following a single illusion induction, the authors regularly interrupted the stroking to measure the recalibration. Interestingly, the authors found that this type of manipulation made participants’ recalibration occur not only during synchronous stroking, but also during asynchronous stroking. However, the self-reports remained stable throughout the experiment, suggesting that the sense of body ownership and recalibration of hand position are supported by different mechanisms of multisensory integration.

Furthermore, in Experiment 1 we were also able to replicate the findings from the study of Tajadura-Jimenez and colleagues^36^, using a different procedure, i.e., the experimenter pulled and pressed the participant’s finger instead of having the participant doing it. This suggests that it is the pulling and pressing *per se*, associated with the sound that provides the sense of elongation. Indeed, one could have claimed that the results of Tajadura-Jimenez and colleagues^36^ may only apply to contexts in which the participants pull or press their own finger, suggesting the involvement of sense of agency. On the contrary, our results integrate the ones by Tajadura-Jimenez and colleagues^36^ suggesting that any pressure or touch is sufficient to create the association of an arbitrary sound with one’s body and elicit the illusion; therefore, our results suggest that agency over the movement is not a necessary component of this illusion. The finding differs from those with other bodily illusions involving sound and touch in which the feelings of agency over the action are necessary to observe changes in body representation (e.g., the illusion of “arm elongation”, see Tajadura-Jiménez *et al*., 2015). Research on the ‘rubber-hand-illusion’ has shown contradictory results on whether active-touch, as opposed to passive-touch, may enhance the illusion^45,51–53^. Nevertheless, findings from another bodily illusion not involving visual cues, but only tactile and proprioceptive cues (the “numbness illusion”, in which participants stroking their own finger and the experimenter’s finger results in a disruption of tactile sensation and body-ownership over one’s own finger), showed that similar feelings of body-ownership over a finger being touched arises similarly during both active- and passive-touch conditions as long as tactile input to the participant is maintained^54^. This study supports our suggestion that the illusion similarly arise during active- and passive-touch conditions since in both types of conditions participants always receive the same tactile input.

Interestingly, and consistent with the findings from Tajadura-Jimenez and colleagues^36^, neither adults nor children reported the sense of having the finger shorter and did not recalibrate their finger sense position. The fact that both groups only reported the illusion of elongation and not shortening suggests that top-down factors, such as experience seeing one owns body growing, may have constrained multisensory integration mechanisms commonly at play for the perception of the illusion. In other words, both children and adults experience that the body and its single parts expand throughout development but do not shrink.

This explanation seems to be corroborated by other studies that have manipulated body size to observe how it effects sense of ownership or tactile judgments. For instance, Pavani and Zampini^11^ found that adults exposed to a rubber-hand-like illusion only recalibrated their felt hand position when the size of the hand was real or enlarged, but not when it was smaller. Other studies involving seeing an enlarged or shrunken image of one’s hand have also failed to elicit illusions of body shrinkage as opposed to those illusions of body expansion^55,56^. Tajadura-Jimenez and colleagues^22^ were able to induce the feeling of being taller by artificially lengthening the time it takes to hear the impact of an object being dropped from one’s hand on the ground but failed to induce the feeling of being shorter with this manipulation. In a similar vein, de Vignemont and colleagues^17^ found that tactile distances feel larger when the stimulated body part feels elongated (following tendon vibration). On the contrary, illusory finger shrinkage did not modulate tactile distance. While some studies have managed to induce illusory body shrinkage^57–59^, there are many fewer reports in previous literature of illusions of body shrinkage than of body expansion, suggesting that the former may be more difficult to elicit than the latter.

Taken together, the results of Experiment 1 reveal that the notion of the body image may be well in place by age 4 and facilitates or constrains online the perception of elongation or shortening of the finger – respectively. Furthermore, recalibration of sense of finger position likely requires multisensory integration abilities that children do not possess by age 4–6, which is in line with previous studies conducted in the same age range^8,26,45^. The results from our study extend this notion to audio-tactile processing, suggesting that, even if children have proved to display auditory dominance in other multisensory tasks^43^, this dominance may not be sufficient in driving the sensitivity to the illusion. It should also be noted that, in both experiments, children appeared to be more sensitive to the tactile rather than the auditory modality, which is in line with the study of Petrini and colleagues^32^, in which they found that children of similar age weight more the haptic than the auditory information when judging the size of objects.

Results from Experiment 2 showed that neither adults nor children were sensitive to the illusion when the axis was shifted from vertical to horizontal. Indeed, both groups did not report the illusion, and did not present recalibration. The lack of the illusion in Experiment 2 supports our hypothesis that crossmodal ‘metaphoric’ correspondences influence the binding of multisensory information, in turn reversing the effects of the illusion. Indeed, previous research has highlighted that high pitches tend to be mapped to high positions in space, while low pitches tend to be mapped to low positions in space across different domains^43,60^, and our results add new evidence to this notion, by extending it to bodily illusions.

It should be noted that Rusconi and colleagues^43^ found that pitch is also mapped on a horizontal dimension in expert musicians (particularly pianist), which suggests that maybe musicians could prove sensitive to the auditory Pinocchio illusion even on the horizontal axis.

In conclusion, in this study we have shown that children and adults are differently susceptible to the auditory Pinocchio illusion depending on axis (vertical vs horizontal): while they both reported feelings of elongation when the pulling of the finger was associated with an ascending sound, they did not experience the same illusion when the same audio-tactile pairing was applied to their finger along a horizontal axis. Thus, our study reveals that the representation of the body is not as malleable as commonly thought, and that top-down and higher-order representations of sensory cues may prevent the body from adapting.

Finally, the possible neurodevelopmental implications of this study should be remarked upon. From a theoretical point of view, our study expands previous studies showing that multisensory mechanisms are foundational to the development of bodily representation, and this extends also to audio-tactile interactions, which have not been investigated in children so far. Furthermore, the fact that children reported the illusion as adults did, reveals that at least multisensory interactions that characterise the body image are already adult-like by age 4. This in turn has clinical implications, as recent studies have shown that therapeutic interventions, aimed at ameliorating the cognitive and social development of children suffering from a variety of neurodevelopmental disorders, including autism and attention-deficit hyperactivity disorder, capitalise precisely on multisensory processing. Indeed, it has been shown that children with autism, for example, present altered multisensory temporal binding windows^59,61,62^, as well as impaired body perception^63^. However, using multisensory interventions, such as whole-body interactions and auditory feedback, appear to promote better sensory processing, better attention skills, and better socialisation skills^64^. Thus, future studies could use multisensory body illusions in the developmental atypical population to better understand the impaired mechanisms and promote new therapies.

At last, we would like to highlight some limits of our study. First, in Experiment 2, the relative position of the two hands was different in comparison to Experiment 1. In other words, while performing the pointing, participants could have crossed their hands which, in turn, changes the external reference frame. This might have influenced limb localisation and consequently their ability to spatially localise them. Indeed, several studies have shown that not only adults, but also infants and children exhibit larger localisation errors when asked to localise touch with crossed limbs^47,48,65^. To control for this possibility, a possible future study could make use of a third axis and have participants place their left hand on the table with fingers pointing rightward, and then ask to point at their middle finger from beneath the table to avoid crossing hands.

Another limit of the study is that we only tested 4 to 6-year-old children in our study. Indeed, a broader inclusion of different age groups could have provided a better view into the developmental trajectories of bodily illusions.

Finally, to be able to draw more robust conclusion about the involvement of multisensory interactions in the development and constrains to body illusions, future studies should integrate multisensory integration tasks when assessing body illusions in children, to observe whether individual differences in multisensory integration abilities can predict performance on body illusions^66–68^.

## Supplementary information

Supplementary Dataset 1.

## Data Availability

All data generated or analysed during this study are included in the Supplementary Material.
