# Supplementary material for: Auditory-induced body distortions in children and adults
Source: Sci Rep. 2020 Feb 20;10:3024. doi: 10.1038/s41598-020-59979-0 (PMC7033089; doi:10.1038/s41598-020-59979-0)
Supplement: Supplementary file 1 — Supplementary Dataset 1. [file 41598_2020_59979_MOESM1_ESM.pdf]

Supplementary dataset

Auditory-induced body distortions in children and adults

Elena Nava & Ana Tajadura-Jimenez

Table 1. Experiment 1: Pointing

Group 1 = Adults
Group 2 = Children

Sound	ASCENDING		DESCENDING		CONSTANT		
Finger	**Pulling**	**Pushing**	**Pulling**	**Pushing**	**Pulling**	**Pushing**	**Baseline pointing (no sound)**
**GROUP**							
1	3,00	-1,50	1,60	-0,20	0,00	-2,50	2,10
1	1,00	0,00	-0,20	-0,40	0,80	0,60	1,20
1	1,00	0,40	2,60	-2,00	1,30	-1,00	1,20
1	1,90	-1,00	0,00	-1,50	0,30	-0,40	-0,40
1	0,20	-2,50	-2,20	-2,60	-0,20	-2,20	2,60
1	1,40	-0,30	-1,60	-1,60	-1,60	-1,40	1,80
1	0,60	-0,60	0,80	-0,30	0,80	-2,20	2,40
1	3,30	1,00	2,70	-2,00	1,00	-1,70	3,40
1	-0,20	-1,90	-0,80	-2,60	-0,90	-1,20	-1,40
1	5,90	-2,80	1,60	-2,20	1,70	-1,70	0,00
1	7,00	0,30	0,20	-2,40	1,60	1,00	0,60
1	5,60	2,20	1,80	-2,20	1,20	-0,40	2,10
1	2,80	-1,00	-1,40	-2,00	0,80	0,00	-0,20
1	4,60	1,20	1,20	-0,20	1,00	0,00	2,00
1	3,50	0,60	1,40	-1,80	1,80	-0,40	1,00
1	2,60	1,60	1,20	-0,40	1,70	0,20	1,20
1	4,00	-0,80	0,00	-0,20	0,80	0,20	-0,20
1	5,40	1,20	1,20	0,20	1,40	0,00	1,70
1	1,80	-0,20	0,40	-1,00	0,80	-0,20	0,40
1	1,00	0,40	0,40	-0,80	1,20	-0,40	1,00
1	1,40	0,40	-0,60	0,00	0,20	-1,80	-3,80
1	-1,40	-4,00	-1,40	-3,80	-0,20	-2,40	0,60
1	3,20	2,00	2,80	1,80	2,40	2,60	1,40
1	1,20	1,20	2,40	0,80	2,20	0,20	-2,20
1	-0,40	-1,20	-1,20	-0,20	-0,20	-0,20	-2,40
1	3,80	2,40	4,00	1,20	4,00	1,80	2,60
1	2,60	1,60	2,80	3,00	3,20	2,40	2,60
1	-1,60	-3,20	-1,80	-3,00	-1,80	-3,60	-1,40
1	2,20	-0,50	-0,30	-2,10	2,50	-0,80	1,20
1	5,00	2,20	3,80	1,60	4,20	1,20	1,80
Mean	**2,41**	**-0,09**	**0,71**	**-0,90**	**1,07**	**-0,48**	**0,76**
St. Dev.	**2,16**	**1,68**	**1,70**	**1,57**	**1,40**	**1,45**	**1,68**
2	2,50	-1,20	-2,70	-7,00	3,10	-4,60	-2,00
2	0,40	-1,00	1,20	-4,00	0,60	-0,80	2,70
2	3,90	7,60	1,70	-5,10	2,10	-3,10	6,60
2	1,60	-4,40	3,20	0,20	-0,80	1,20	-3,70
2	-3,00	-2,50	0,40	-0,40	1,60	-1,50	-0,70
2	3,10	-4,00	2,60	-7,30	4,70	-3,40	0,90
2	3,10	-9,10	-4,40	-6,80	4,10	-8,60	-1,70
2	-2,60	5,30	-0,40	6,60	3,70	5,00	2,40
2	-3,20	-1,60	-2,10	-4,00	2,90	-2,30	-1,40
2	4,20	2,00	3,20	0,40	5,60	2,60	3,80
2	-4,80	-1,50	-5,20	-5,80	6,20	-3,80	2,80
2	-2,40	-5,40	-3,20	-6,20	5,00	-7,70	-0,50
2	1,20	-7,60	5,00	-2,60	4,00	-1,80	2,00
2	-1,60	-4,80	1,00	-6,00	-1,00	-4,20	-2,60
2	3,20	2,60	4,20	-4,80	2,60	2,20	2,60
2	0,67	-5,60	5,60	-7,40	0,20	-4,20	-1,20
2	-1,00	-3,20	-4,20	-6,60	-3,00	-3,80	-0,60
2	7,60	-2,80	8,00	-2,40	7,40	-2,20	-0,60
2	-3,60	-5,80	-3,00	-6,80	-4,00	-6,40	-1,20
2	7,20	7,60	7,40	7,00	7,20	7,00	6,80
2	-4,20	-0,20	0,20	-3,40	3,40	-3,00	-2,40
2	-2,40	-2,40	-2,20	-2,60	-2,40	-2,20	-2,40
2	-1,60	-2,20	0,40	-2,40	-2,80	-7,80	-4,20
2	-0,20	-4,00	-0,20	-1,80	0,20	-4,20	-0,60
2	2,40	2,40	0,00	11,60	1,80	1,40	1,00
2	6,80	8,60	7,40	10,40	9,00	7,60	5,80
2	7,60	-6,00	8,60	-4,40	2,40	-5,70	3,70
2	5,80	-8,00	4,40	-5,40	2,60	-8,20	-4,60
2	9,40	9,80	4,60	10,20	7,80	8,60	3,80
2	2,00	-10,00	7,00	6,40	0,60	-9,60	-2,60
Mean	**1,40**	**-1,58**	**1,62**	**-1,68**	**2,49**	**-2,12**	**0,40**
St. Dev.	**3,97**	**5,27**	**4,01**	**5,77**	**3,35**	**4,80**	**3,14**

Table 2. Experiment 2: Pointing

Sound	ASCENDING		DESCENDING		CONSTANT		
Finger	**Pulling**	**Pushing**	**Pulling**	**Pushing**	**Pulling**	**Pushing**	**Baseline pointing (no sound)**
**GROUP**							
1	0,00	-0,40	1,40	0,40	1,60	-0,40	-0,60
1	0,30	-0,60	1,40	-3,60	-1,00	2,40	0,40
1	2,80	2,10	-2,30	3,50	-0,20	-2,00	-2,00
1	0,30	-0,70	-2,20	-1,00	-0,70	0,70	-2,00
1	0,30	-0,70	1,30	-1,30	-0,30	-0,70	-2,60
1	2,50	0,50	2,60	-3,40	1,80	-0,70	1,70
1	4,60	-0,20	3,40	-0,60	-3,50	-0,20	0,80
1	1,40	0,80	0,80	0,70	1,80	0,80	-4,80
1	1,00	-0,50	0,60	-0,60	-0,80	1,40	0,40
1	-0,20	-0,80	-2,10	0,00	-0,90	0,40	-0,60
1	-1,30	-0,60	-1,40	-1,30	-2,30	-0,10	-4,40
1	1,10	0,30	0,00	1,10	0,50	-0,10	2,90
1	1,70	-1,60	0,80	-1,70	1,00	-0,80	2,00
1	1,67	-1,00	1,33	-2,00	1,33	-0,80	0,00
1	1,50	-1,25	0,60	-1,75	0,00	-1,00	0,25
1	0,90	-0,80	0,80	-3,60	-0,80	-2,00	0,40
1	1,50	-1,25	0,60	-2,67	0,33	-1,00	0,25
1	1,50	-1,00	1,33	-2,20	1,50	-0,80	-0,25
1	4,60	-0,50	3,00	-1,40	2,33	-0,20	0,25
1	3,80	1,60	4,20	2,20	4,00	3,00	0,40
1	3,80	1,20	4,00	2,80	3,20	2,80	1,20
1	0,00	-0,80	0,20	-1,40	-0,20	-1,00	-1,60
1	0,80	-1,00	0,60	-0,80	0,60	-0,60	0,80
1	5,40	-5,40	4,00	-6,40	3,20	-2,80	-1,60
1	4,00	2,60	4,60	3,00	5,00	3,80	2,40
1	2,00	-0,20	2,00	-1,20	1,40	0,20	3,60
1	-0,40	-0,60	-0,40	-0,40	-0,20	-0,60	-0,40
1	3,20	-1,40	2,20	-3,40	1,60	-3,00	0,40
1	6,40	-7,80	1,30	0,40	2,30	0,30	1,00
1	1,80	-5,50	2,40	-1,60	0,90	0,00	-4,00
Mean	**1,90**	**-0,85**	**1,24**	**-0,94**	**0,78**	**-0,10**	**-0,19**
St. Dev.	**1,87**	**2,12**	**1,84**	**2,16**	**1,81**	**1,57**	**2,01**
2	2,50	-1,90	6,30	-3,10	2,20	-2,30	-0,30
2	2,50	1,20	3,20	0,00	2,20	0,80	2,50
2	1,50	-1,60	1,40	2,20	7,90	-3,80	1,20
2	-2,60	-3,60	1,40	-2,20	-1,40	-2,40	0,50
2	1,10	-2,40	-2,20	-4,40	-1,00	-2,90	1,80
2	2,70	-0,90	1,40	-1,50	2,00	-0,30	1,20
2	5,90	2,90	4,10	0,10	4,30	-1,00	3,30
2	6,60	4,30	5,70	3,10	1,80	4,50	4,20
2	-0,30	1,10	-0,50	-0,80	2,50	-0,10	-2,90
2	1,60	-0,80	1,30	-2,80	0,30	-3,40	-0,60
2	-0,70	-3,20	-2,00	-3,30	-0,20	-2,80	1,10
2	2,40	0,50	0,80	0,00	3,20	0,00	0,60
2	0,70	-2,60	1,40	-6,00	2,80	-7,00	-2,80
2	6,80	-1,20	-0,40	-8,20	-3,20	-6,40	0,40
2	0,00	-1,50	0,70	-3,20	-0,90	-1,50	-0,80
2	3,90	-0,60	0,40	-0,50	1,70	0,20	-0,20
2	4,90	0,50	0,80	-1,40	3,10	-1,40	0,20
2	2,50	-3,70	-4,30	-8,20	-2,90	-4,80	-1,40
2	0,50	-3,00	1,90	-2,80	3,50	-1,20	2,00
2	5,80	5,80	6,60	6,40	5,80	4,80	4,00
2	-1,80	-1,00	0,00	2,20	2,40	1,20	4,40
2	2,20	2,20	2,40	3,80	0,20	3,20	4,40
2	7,80	-1,40	10,00	0,00	9,20	1,00	4,40
2	3,60	3,40	3,40	3,40	-0,80	3,80	0,60
2	4,40	3,20	7,60	2,80	5,40	3,20	0,60
2	5,60	6,00	7,20	5,60	7,60	3,60	6,60
2	7,60	3,00	5,70	2,30	7,50	2,30	-6,20
2	5,80	3,60	7,80	6,40	8,20	7,40	9,00
2	8,60	7,60	8,00	6,80	8,20	7,80	5,80
2	12,00	-5,60	12,00	-8,80	8,00	0,20	-0,40
Mean	**3,47**	**0,34**	**3,07**	**-0,40**	**2,99**	**0,09**	**1,44**
St. Dev.	**3,34**	**3,25**	**3,86**	**4,30**	**3,56**	**3,66**	**3,08**

Group 1 = Adults
Group 2 = Childre

Table 3. Experiment 1: Questionnaire pulling

	Sound ascending - Finger pull		Sound descending - Finger pull		Sound constant - Finger pull	
GROUP	I felt my finger longer	I felt my finger shorter	I felt my finger longer	I felt my finger shorter	I felt my finger longer	I felt my finger shorter
1	1	-2	0	-2	0	-2
1	1	-2	1	-1	0	-2
1	1	-2	0	-2	0	0
1	1	-2	1	-2	0	-2
1	1	-2	1	-2	0	-2
1	1	-2	1	-2	0	-2
1	1	-2	1	-2	0	-2
1	1	-2	1	-2	0	0
1	1	-2	0	-2	0	-2
1	2	-2	1	0	-2	-2
1	2	-2	1	-2	-1	-1
1	1	-1	1	-1	-1	-2
1	1	-2	1	-2	-1	-2
1	1	-2	-1	-2	0	-2
1	0	-2	-1	-1	-2	-2
1	2	-2	-2	-2	-1	-2
1	1	-2	-1	1	0	0
1	2	-2	2	-2	0	-2
1	2	-2	-2	-1	-1	-2
1	2	-1	1	0	-2	-2
1	-2	-2	-2	-2	-2	-2
1	1	-2	-2	1	-2	-2
1	2	-2	-2	-2	1	-2
1	-2	-2	-2	2	2	-2
1	1	-2	-2	1	1	-2
1	2	-2	-2	2	2	-2
1	-2	-2	-2	1	-2	-2
1	2	-2	-2	1	2	-2
1	-1	-2	-2	-2	-1	-2
1	1	-2	-2	2	1	-2
Mean	**0,90**	**-1,93**	**-0,47**	**-0,83**	**-0,30**	**-1,77**
St. Dev.	**1,18**	**0,25**	**1,43**	**1,49**	**1,21**	**0,63**
2	2	-2	2	0	-1	-1
2	-1	-2	-2	-2	0	1
2	0	1	-2	-2	-2	-2
2	1	2	2	0	-1	-1
2	2	0	-2	-2	-2	-2
2	2	-1	1	1	-1	-1
2	2	-2	1	-2	0	-2
2	1	1	-2	-2	-2	-2
2	2	1	-2	-2	-1	-2
2	2	0	-2	-2	-2	-2
2	2	-2	1	-2	-1	-2
2	2	1	1	-2	0	-2
2	2	0	-2	-2	-2	-2
2	2	0	1	-2	-1	-2
2	1	1	-2	-2	-2	-2
2	2	1	-2	-2	-1	-2
2	2	-2	1	-2	1	-2
2	2	-2	1	-2	1	-2
2	2	-2	-2	2	2	-2
2	2	-1	-2	1	-2	-2
2	-2	-2	-2	-1	-2	-1
2	2	-2	2	-2	-1	-2
2	-2	1	-2	2	2	-2
2	-2	-1	-1	-2	1	-2
2	2	-2	-1	-2	2	-2
2	-1	-2	-2	1	0	0
2	2	-2	2	-2	1	-2
2	1	-2	-1	-2	1	-2
2	2	-2	-2	-1	1	-2
2	2	-2	-2	1	0	0
Mean	**1,20**	**-0,80**	**-0,67**	**-1,13**	**-0,40**	**-1,63**
St. Dev.	**1,37**	**1,37**	**1,63**	**1,38**	**1,35**	**0,76**

Group 1 = Adults
Group 2 = Children

Table 4. Experiment 1: questionnaire pressing

Group 1 = Adults
Group 2 = Children

	Sound descending - Finger push		Sound Ascending - Finger push		Sound constant - Finger push	
GROUP	I felt my finger longer	I felt my finger shorter	I felt my finger longer	I felt my finger shorter	I felt my finger longer	I felt my finger shorter
1	-2	-1	-2	-2	-1	0
1	-2	-1	-2	-2	-2	-2
1	-2	-2	-2	-2	-2	-2
1	-1	-1	-1	-2	-2	1
1	-2	-2	-2	-2	-2	-2
1	-2	1	-2	1	-2	1
1	-2	1	-2	1	-2	-2
1	-2	-2	-2	-2	0	0
1	-2	-1	-2	-2	0	2
1	0	1	0	1	0	0
1	-2	-2	-2	-2	-2	1
1	-2	-2	-2	-2	-2	0
1	-2	1	-2	1	-2	-1
1	-2	-2	-2	-2	-2	-2
1	-2	-2	-2	-2	-2	-2
1	-2	1	-2	-2	-2	-2
1	-2	1	0	1	-2	0
1	-2	2	-1	1	-2	0
1	-1	1	-2	-2	-1	-1
1	-1	0	-2	1	-2	0
1	-2	-2	-2	-2	-2	-2
1	-2	1	-2	1	-2	1
1	-2	2	-2	-1	-2	-2
1	-2	2	-2	2	-2	2
1	-2	-2	-1	-2	-2	1
1	-2	2	-2	1	-2	2
1	-2	-2	-2	-2	-2	1
1	-2	1	-2	1	-2	1
1	-2	-2	-2	-2	-2	-2
1	-2	2	-2	2	-2	2
Mean	**-1,83**	**-0,23**	**-1,77**	**-0,70**	**-1,73**	**-0,23**
St. Dev.	**0,46**	**1,61**	**0,57**	**1,58**	**0,64**	**1,48**
2	-2	1	-2	1	0	0
2	-2	-1	1	-1	-2	-2
2	-2	1	2	1	-2	1
2	-2	1	-2	1	-2	1
2	-2	1	1	2	-2	1
2	-2	1	-2	1	-2	1
2	-2	1	0	1	-2	1
2	-2	1	2	1	-2	-2
2	-2	-2	1	-2	1	-2
2	-2	1	-2	1	-2	1
2	-2	-2	2	0	-2	1
2	-2	-2	1	-2	1	-2
2	-2	2	0	0	-2	2
2	-2	1	0	1	-2	1
2	-2	1	2	1	-2	-2
2	-2	1	-2	1	-2	1
2	-2	1	2	2	-2	-2
2	-2	2	-2	2	-2	1
2	-2	-2	-2	1	2	-2
2	-2	1	-2	-1	-2	1
2	-2	2	2	-2	-2	1
2	0	0	-2	2	0	0
2	-2	1	-1	-2	0	0
2	-1	-2	-1	-2	0	0
2	0	0	-2	1	0	0
2	-1	-2	2	-2	-1	-2
2	-2	-1	-1	-2	-2	-2
2	-2	-2	-2	-1	0	0
2	0	0	-2	1	0	0
Mean	**-1,72**	**0,14**	**-0,31**	**0,14**	**-1,14**	**-0,14**
St. Dev.	**0,65**	**1,41**	**1,69**	**1,46**	**1,22**	**1,36**

Table 5. Experiment 2: questionnaire pulling

Group 1 = Adults
Group 2 = Children

	Sound ascending - Finger pull		Sound descending - Finger pull		Sound constant - Finger pull	
**GROUP**	**I felt my finger longer**	**I felt my finger shorter**	**I felt my finger longer**	**I felt my finger shorter**	**I felt my finger longer**	**I felt my finger shorter**
1	1	-2	1	-2	-2	1
1	1	-2	0	-1	0	-2
1	1	-2	0	-2	-2	-2
1	2	-2	1	-2	0	0
1	-1	-2	-1	-2	0	-1
1	1	-2	-2	-2	1	-2
1	2	-2	-2	-2	1	-2
1	1	-2	0	-2	-2	-2
1	1	-2	1	-2	-2	1
1	1	-2	-1	-2	-2	-2
1	-1	-2	1	-2	0	-2
1	1	-2	1	-2	-2	1
1	-1	-2	1	-2	0	-2
1	1	-2	-1	-2	-2	-2
1	1	-2	0	-2	0	1
1	1	-2	-2	-2	-2	-2
1	2	-2	1	-2	0	-2
1	1	-2	0	-2	-2	-2
1	1	-2	0	-1	0	-2
1	-2	-2	-2	-2	-2	-2
1	-2	-2	-2	-2	-2	-2
1	-2	-2	-2	-2	-2	-2
1	-2	-2	-2	-1	-2	-2
1	1	-2	-2	-1	-1	-2
1	-2	-2	-2	1	-2	-2
1	1	-2	-2	1	-1	-2
1	-2	-2	-2	-2	-2	-2
1	1	-2	-2	1	-1	-2
1	1	-1	1	-1	0	-1
1	1	-2	-2	-2	-2	-2
Mean	**0,30**	**-1,97**	**-0,70**	**-1,53**	**-1,10**	**-1,47**
St. Dev.	**1,37**	**0,18**	**1,29**	**0,94**	**1,06**	**1,07**
2	1	0	1	-2	1	-1
2	1	-2	-2	-2	1	-2
2	2	1	1	-2	1	-2
2	1	1	-2	-2	-2	-2
2	0	-2	1	-2	1	-2
2	1	-2	1	-2	1	-2
2	0	0	-2	-2	0	0
2	2	1	0	0	1	-2
2	1	1	1	-2	2	1
2	2	-2	2	1	1	-2
2	2	-2	1	-2	1	-2
2	2	-2	-2	-2	1	-2
2	1	1	1	-2	-2	-2
2	2	-2	1	-2	1	-2
2	2	-2	-2	-2	1	-2
2	2	-2	1	-2	1	-2
2	2	-2	1	-2	1	-2
2	2	-2	1	-2	1	-2
2	2	1	-2	-2	1	-2
2	-2	-2	-2	1	-2	-1
2	0	0	0	0	0	0
2	-2	-2	-2	-2	-2	-2
2	2	-2	-1	-2	1	-2
2	0	0	2	-2	2	-2
2	0	0	0	0	0	0
2	1	1	1	-2	-2	-2
2	-2	-2	-2	-2	-2	-2
2	0	0	0	0	0	0
2	1	-2	1	-2	1	-2
2	2	-2	-2	2	-1	-2
Mean	**0,93**	**-0,90**	**-0,13**	**-1,40**	**0,27**	**-1,57**
St. Dev.	**1,26**	**1,32**	**1,46**	**1,16**	**1,28**	**0,86**

Table 6. Experiment 2: questionnaire pressing

	Sound descending - Finger push		Sound ascending - Finger push		Sound constant - Finger push	
GROUP	I felt my finger longer	I felt my finger shorter	I felt my finger longer	I felt my finger shorter	I felt my finger longer	I felt my finger shorter
1	-2	1	0	1	-2	-2
1	-2	1	-2	1	-2	0
1	-2	1	-2	1	-2	-2
1	-2	1	-2	1	-2	-2
1	-2	1	-2	-2	-2	-2
1	-2	1	-2	1	-2	-2
1	-2	1	-2	1	-2	-2
1	-1	1	-2	1	-2	-2
1	-2	1	0	1	-2	-2
1	-2	1	-2	1	-2	0
1	-2	1	-2	-1	-2	1
1	-2	1	0	1	-2	-2
1	-2	1	-1	-1	-2	1
1	-2	1	-2	1	-2	0
1	-2	1	-2	1	-2	0
1	-2	1	-2	1	-2	-2
1	-2	1	-2	1	-2	-2
1	-2	1	-2	1	-2	-2
1	-2	1	-2	1	-2	0
1	-2	-2	-2	-2	-2	-2
1	-2	-2	-2	-2	-2	-2
1	-2	-2	-2	-2	-2	-2
1	-2	-2	-2	-1	-2	-1
1	-2	-1	-2	2	-2	-1
1	-2	1	-2	-2	-2	-2
1	-2	1	-2	-2	-2	-1
1	-2	-2	-2	-2	-2	-2
1	-2	1	-2	-1	-2	-1
1	-2	2	-2	1	-2	1
1	-2	-2	-2	1	-2	-2
Mean	**-1,97**	**0,37**	**-1,77**	**0,07**	**-2,00**	**-1,23**
St. Dev.	**0,18**	**1,27**	**0,63**	**1,36**	**0,00**	**1,07**
2	-2	1	1	1	-2	1
2	-2	-1	-2	-1	-2	-1
2	1	2	-2	1	-2	1
2	-2	1	-2	1	-2	-2
2	-2	1	1	1	-2	1
2	-1	-1	1	1	-2	-1
2	-2	2	-2	-1	-2	1
2	1	2	0	0	-2	1
2	2	1	-2	1	2	1
2	-2	2	2	1	-2	1
2	-2	2	-2	1	-2	1
2	-1	2	-2	1	-2	1
2	-2	1	-2	1	-2	-2
2	-2	2	1	1	-2	1
2	-1	2	-2	1	-2	1
2	-2	2	-2	1	-2	1
2	-2	2	-2	1	-2	1
2	-2	2	-2	1	-2	1
2	1	2	-2	1	-2	1
2	-2	-2	0	0	0	0
2	0	0	0	0	0	0
2	-2	-2	-2	-2	-2	-2
2	-2	2	-2	1	0	0
2	-2	1	-2	2	-2	1
2	0	0	0	0	0	0
2	-2	1	1	1	-2	-2
2	-2	-2	-2	-2	-2	-2
2	0	0	0	0	0	0
2	-2	-1	-2	-1	-2	-1
2	-2	1	0	0	0	0
Mean	**-1,27**	**0,83**	**-0,97**	**0,43**	**-1,47**	**0,10**
St. Dev.	**1,20**	**1,37**	**1,35**	**0,97**	**1,04**	**1,16**

Group 1 = Adults
Group 2 = Children
